# Prevention of Sickness under the Act

**Published:** 1914-06-06

**Authors:** 


### NATIONAL INSURANCE ACT DEVELOPMENTS.

### Prevention of Sickness under the Act.

In spite of the fact that the National Insurance Act has now been in operation for nearly two years, there are certain of its provisions which, up to the present time, have received little or no attention. The full title of the Act of 1911 is "An Act to provide for insurance against loss of health, and for the prevention and cure of sickness, and for insurance against unemployment, and for purposes incidental thereto." Attention has so far been directed almost exclusively to the insurance side of the Act—that is to say, to the payment of benefits when sickness arises; and very little regard has been had to the equally important—or perhaps more important—provisions which relate to the prevention of sickness.

It has been stated with some authority that sickness claims on the Approved Societies in certain districts and occupations, and particularly among married women, have proved abnormally heavy. Though the truth of this statement is not yet fully demonstrated, and though the extent of the excess is not known, yet the fact is admitted by the Government, as expressed by the Chancellor of the Exchequer in the provision of the further State aid indicated in his Budget speech.

Now, it is clear that there are two ways of meeting these excess claims. The first is by the direct method of further State subsidies to the societies, while the second is to take appropriate steps to prevent the sickness which now gives the right to claim benefits. It will at once be admitted that if prevention is possible, it is far better from the point of view of the public health, and, in fact, from every point of view, that steps should be taken to make the prevention effective.

### The Powers Conferred by the Act.

It is, therefore, our object to call attention to the powers already conferred by the National Insurance Act for the purpose of dealing with this important question, and we feel sure that if public interest could be aroused to the urgency of the situation, those who are invested with authority to administer the Act would very quickly take steps to put the provisions into operation.

The principal section of the Act of 1911 which deals with the matter is Section 63, and its purport is that if any approved society, Insurance Committee, or the Insurance Commissioners have evidence that the sickness which has taken place among insured persons is excessive, and that such excess is due to some preventable cause, such as bad conditions of employment, bad housing, insanitary conditions in the locality, contaminated water supply, neglect of the enforcement of public health precautions, etc., the Society, Committee, or Commissioners may claim from the person or authority alleged to be in default any extra expenditure incurred in benefit payments by reason of such cause or causes.

It is thus clear that as soon as evidence can be produced that there is an actual excess of sickness due to preventable causes, action may be taken in the matter, not only by the Approved Societies, but also by the Insurance Committees and the Insurance Commissioners.

### Section 63 and Preventable Causes.

That there will be practical difficulties in the application of the powers thus conferred is certain, because it is patent that the relation of cause and effect is difficult to prove in the case of sickness. At the same time these difficulties should not be insurmountable, and they were at least to some extent foreseen by the framers of the Act, since it is enacted that, should it be found impossible to arrive at any agreement on the subject, the Society, Committee, or Commissioners making the claim may apply to the Secretary of State or the Local Government Board for an inquiry, and the latter may appoint a competent person to hold such inquiry. The person thus appointed has all the powers possessed by an inspector of the Local Government Board for the purposes of an inquiry under the Public Health Acts, and has power to order how and by what parties costs are to be paid. It is interesting to note that "a Society or Committee must not be ordered to pay the costs of the other party to the inquiry if the person holding the inquiry certifies that the demand for an inquiry was reasonable under the circumstances; and when he so certifies, the Treasury may repay to the Society or Committee the whole, or any part of, the costs incurred by it."

Should the person holding the inquiry find that during a period of not less than three years before the date of the inquiry (or in the case of an epidemic during any less period) the sickness experienced has exceeded that expected by more than 10 per cent., and that such excess was due, in whole or in part, to any of the causes mentioned above, he may order the extra expenditure on sickness claims, or other benefits, to be made good by the person or authority through whose action or neglect the excess sickness was caused.

Thus, where the excess sickness is due to conditions of employment, the employer is made liable; or where due to bad housing or insanitary conditions, the local authority is to be held responsible in the first place, and then the owner or lessee of the premises. With regard to excess sickness arising from the conditions of employment, it must be clearly borne in mind that any sickness in respect of which compensation is payable by the employer, cannot be, and is not to be, taken into account, and that, furthermore, it must be presumed that employers are only liable when the excess sickness is due to some cause which it is in their power to prevent. If the section were interpreted in any other way it would press unduly hardly upon employers in dangerous trades, as these would in effect be called upon to pay a higher sickness contribution than the average employer, while the occupational risk for which they would be paying is already provided for in the normal contribution. It may be remarked in passing that, although we suggest this as an equitable interpretation, the legal interpretation would probably be in the opposite direction. The wording of the sub-section conferring power to recover from the employer is wide enough to impose liability irrespective of any default on the part of the employer.

It will be noticed that extra expenditure upon sickness claims is only recoverable if the amount of sickness experienced has exceeded that expected by more than 10 per cent., but it should be borne in mind that the amount that may be recovered is the whole excess and not merely such part as is over 10 per cent.; the 10 per cent. limit was probably imposed because this was the original margin for contingencies retained in the contributions charged. Further details as to how claims are to be made for repayment of excessive expenditure, and of the mode of dealing with any sums so recovered, do not call for comment at the moment.

### Why are the Powers Unused?

It may be taken for granted, as before indicated, that the main object of the inclusion of the section in the Act was to institute some means of preventing accident and disease; it is hardly likely that it was the intention of the framers of the Act that having once traced the cause of excessive sickness and penalised the offending person, the cause should remain unremedied. An effective carrying out of the section would thus be exceedingly beneficial to the persons directly involved, to the members of Approved Societies, and to the community generally.

Good, however, though the general principles are, it is exceedingly doubtful whether the machinery that has been instituted is the best that could be devised for the purpose. Since proceedings may be taken at the instance of Approved Societies, Insurance Committees, or the Insurance Commissioners, no one body is responsible, and there is therefore the usual lack of initiative.

So far as Approved Societies are concerned the net result of the section is to require them not only to administer the benefits of the Acts, but to trace, in certain circumstances, the causes of claims. Before any inquiry can be held Approved Societies must themselves make investigation and formulate charges, and the whole of the trouble and expense of such preliminary investigations must apparently be borne by them.

Approved Societies, both small and large, would naturally hesitate before taking steps in a matter involving so much trouble and having at the best a doubtful issue. Small societies operating in a limited area would not possess the staff necessary to undertake the work, while in the case of large centralised societies operating over the whole of the United Kingdom the work entailed in subdividing the members according to district and occupation, as well as age and sex and scale of benefits, in order to trace the excessive sickness to particular individuals or authorities, would be almost prohibitive.

It must be particularly borne in mind that certain definite individuals have to be charged with causing, or allowing to be caused, the excessive sickness claims, and the difficulty of doing this in the case of a large society will be readily appreciated.

### A Practical Suggestion.

We would suggest that a more effective way of dealing with the matter, especially in view of the fact that the administration of benefits in any one locality and in any one occupation is not confined to a single society, would be to form a specially constructed committee, a department, if need be, of the Insurance Commissioners, to deal entirely with investigations into the causes of sickness. Approved Societies suffering excessive sickness expenditure could then refer to the committee such general information as they may possess regarding the origin of these excessive claims, and the committee would be guided by circumstances as to whether or not a detailed investigation should be undertaken.

If information were received from several societies that a certain locality appeared to be productive of heavier claims than the average, this should be sufficient to warrant further inquiry, and the advantage would be secured that only one investigation would be conducted instead of as many investigations as there were societies concerned. Seeing also that it may be taken for granted that claims for the repayment of excessive sickness expenditure will in the great majority of cases be resisted in the first instance, the committee could with advantage be given the same powers as would be possessed by a person appointed to hold an inquiry according to the section as it at present stands. A considerable amount of time and trouble would in this way be saved. The expenses of the committee could if necessary be met by contributions from the Approved Societies, such contributions being based upon the number of members.

It is absolutely necessary in such an important matter as the prevention of disease and the discovery of its cause that the machinery set up should be not only workable but easily set in motion, and we venture to submit that the centralisation of the power effected by adopting the method we suggest will have good results in this direction.

Approved Societies and Insurance Committees would be much more ready to take steps when the assistance of a body of experts was guaranteed than when the whole of the trouble has to be borne by themselves. Be that as it may, the point that we wish to emphasise is that machinery already exists, and, whether easily workable or not, whether capable of improvement or not, it behoves those to whom the power is given to see that it is set in motion with as little delay as possible.

